# A COVID-19 isolation facility for people experiencing homelessness in Berlin, Germany: a retrospective patient record study

**DOI:** 10.3389/fpubh.2023.1147558

**Published:** 2023-06-06

**Authors:** Merle Hörig, Sarah M. Klaes, Svetlana Krasovski-Nikiforovs, Welmoed van Loon, Lukas Murajda, Rosa C. O. Rodriguez, Christine Schade, Anabell Specht, Gabriela Equihua Martinez, Ruth Zimmermann, Frank P. Mockenhaupt, Joachim Seybold, Andreas K. Lindner, Navina Sarma

**Affiliations:** ^1^Charité Center for Global Health, Institute of International Health, Charité – Universitätsmedizin Berlin, Berlin, Germany; ^2^Berliner Stadtmission, Berlin, Germany; ^3^Health Authority Berlin-Mitte, Berlin, Germany; ^4^Department of Infectious Disease Epidemiology, Robert Koch Institute, Berlin, Germany; ^5^Medical Directorate, Charité – Universitätsmedizin Berlin, Berlin, Germany

**Keywords:** homelessness, homeless shelter, isolation and infection control, COVID-19, SARS-CoV-2, people experiencing homelessness, isolation facility

## Abstract

**Introduction:**

People experiencing homelessness (PEH) are disproportionately affected by the COVID-19 pandemic. For many PEH it is impossible to isolate due to the lack of permanent housing. Therefore, an isolation facility for SARS-CoV-2 positive PEH was opened in Berlin, Germany, in May 2020, offering medical care, opioid and alcohol substitution therapy and social services. This study aimed to assess the needs of the admitted patients and requirements of the facility.

**Materials and methods:**

This was a retrospective patient record study carried out in the isolation facility for PEH in Berlin, from December 2020 to June 2021. We extracted demographic and clinical data including observed psychological distress from records of all PEH tested positive for SARS-CoV-2 by RT-PCR. Data on duration and completion of isolation and the use of the facilities’ services were analyzed. The association of patients’ characteristics with the completion of isolation was assessed by Student’s *t*-test or Fisher’s exact test.

**Results:**

A total of 139 patients were included in the study (89% male, mean age 45 years, 41% with comorbidities, 41% non-German speakers). 81% of patients were symptomatic (median duration 5 days, range 1–26). The median length of stay at the facility was 14 days (range 2–41). Among the patients, 80% had non-COVID-19 related medical conditions, 46% required alcohol substitution and 17% opioid substitution therapy. Three patients were hospitalized due to low oxygen saturation. No deaths occurred. Psychological distress was observed in 20%, and social support services were used by 65% of PEH. The majority (82%) completed the required isolation period according to the health authority’s order. We did not observe a statistically significant association between completion of the isolation period and sociodemographic characteristics.

**Conclusion:**

The specialized facility allowed PEH a high compliance with completion of the isolation period. Medical care, opioid and alcohol substitution, psychological care, language mediation and social support are essential components to address the specific needs of PEH. Besides contributing to infection prevention and control, isolation facilities may allow better access to medical care for SARS-CoV-2 infected PEH with possibly positive effects on the disease course.

## 1. Introduction

People experiencing homelessness (PEH) are particularly affected by the COVID-19 pandemic. Many facilities providing essential services for PEH, such as food distribution sites, community centers and public institutions such as libraries were closed during the pandemic (1). Emergency shelters had to reduce their bed capacity for infection prevention and control (IPC), leaving more people unsheltered (2). Income generating job opportunities were reduced, resulting in an aggravation of precarious financial situations. Social distancing and the lack of specific support might have increased psychosocial stress among PEH, leading to an exacerbation of existing mental health conditions (3). Some reports point out that PEH faced an additional problem of criminalization caused by difficulties in following governmental IPC regulations (3).

Current research indicates an increased risk of SARS-CoV-2 infection among PEH. In France, seroprevalence in PEH was found to be higher than in the general population (4). Crowded living conditions in shelters, sharing sanitary facilities and common areas, sleeping in dormitories, and the difficulty of keeping distance and washing hands regularly contribute to an increased infection risk (4–7). Additionally, frequent changes of location among PEH leads to a high level of fluctuation of shelter residents which possibly contributes to the spread of the virus (6) and makes it especially difficult to trace contacts in order to mitigate transmission (1). Moreover, a generally high proportion of asymptomatic infections increases the risk of non-detection or late detection (8). The increased risk of infection for PEH may vary depending on local conditions and the current course of the pandemic. For instance and in contrast to the above findings from France, a study in Italy found between October 2020 and June 2021 a similar prevalence of SARS-CoV-2 infection among PEH and the general population (9).

PEH are considered particularly vulnerable to develop severe COVID-19 due to an increased prevalence of pre-existing health conditions and risk factors (10). Studies in high income countries found a higher prevalence of HIV, hepatitis C and tuberculosis in PEH than in the general population (11, 12), and hypertension, diabetes, COPD, asthma and other non-communicable diseases are frequent and often poorly controlled (13–16). Mental disorders are also increased among PEH (14, 17, 18), and additional pandemic stressors may worsen pre-existing conditions, such as depression, posttraumatic stress disorder, sleeping disorder, psychosis or substance use disorders (19). Reasons for this overall lower health status are multifactorial and include both individual factors as well as structural factors including access barriers to health care, socioeconomic inequities, harsh life conditions, discrimination, and poor nutrition (14, 20). All of these aspects contribute to the fact that PEH should be considered a vulnerable group in the context of COVID-19.

Berlin has a population of 3,850,809 inhabitants (21). During an official census in February 2020, 1.976 roofless PEH – according to the definition of European Typology of Homelessness and Housing Exclusion (ETHOS) – were counted in Berlin (22). Welfare organizations, however, assume a much higher number of around 6.000 roofless PEH living in Berlin (23). Demographic data on roofless PEH in Berlin is scarce, but according to single studies that were carried out among PEH in and around low threshold outpatient clinics, median age was found to be between 41 and 43 years and the proportion of men was with around 4/5 much higher than the proportion of women (11, 24). PEH without shelter in Berlin are a mobile population. They live all over the city. Accordingly, services for PEH, such as shelters, soup kitchens, day centers, counseling services and low threshold medical centers are spread across Berlin (25). Regarding IPC, quarantine and self-isolation, PEH may face structural barriers and challenges in particular. Following the pandemic onset, a 10-point-action-plan was published by the German national working group on homelessness services (Bundesarbeitsgemeinschaft Wohnungslosenhilfe, BAG W), including the demand for adequate isolation and quarantine opportunities (26), and the Robert Koch Institute (RKI), the German public health institute, emphasized the need for quarantine and isolation facilities tailored to the needs of PEH (27). In May 2020, Berlin’s first isolation and quarantine facility for PEH opened to provide isolation and quarantine possibilities including medical and social care (28).

As this is the first study that analyses patient records of a COVID-19-isolation facility in Germany and since little is known about the specific requirements of such a facility to meet the needs of PEH, we aim to answer the following questions: Did the centralized isolation facility for PEH in Berlin provide a good strategy for completing the isolation period? What kind of services and requirements must such a facility have to meet the needs of the admitted patients?

The present study analyses data from the isolation unit of the isolation and quarantine facility. Hereby we aim to gain a better understanding of the complex needs of PEH for completing the isolation period and give recommendations regarding future projects. We describe the services provided by the facility and their utilization, as well as demographic and clinical parameters of the admitted PEH.

## 2. Methods

### 2.1. Design and setting

This was a retrospective patient record study carried out at a COVID-19 isolation facility for PEH in Berlin. Records of all patients admitted for isolation between December 2020 and June 2021 were assessed. In most cases, patients were referred to the COVID-19 quarantine and isolation facility by one of Berlin’s support services for PEH, such as medical centers, night shelters and soup kitchens, with a positive SARS-CoV-2 rapid antigen test. Upon arrival, they were admitted to the quarantine unit in a single room, where confirmatory RT-PCR testing was performed on site. In case of a confirmed infection, patients were offered cohort isolation in the isolation unit for the required period according to the local health regulations. Admission criteria were set by the local health authority Berlin Mitte and included self-declared homelessness and a positive SARS-CoV-2-test result. Only patients above the age of 18 years were included in this analysis. The number of all admitted patients during the study period with a confirmed SARS-CoV-2 infection above the age of 18 years determined the sample size of the study.

The isolation and quarantine facility was located in the center of Berlin near the main train station on the grounds of the Berlin City Mission (Berliner Stadtmission), an association in Berlin that provides a range of services for PEH. On the same site, there was an outpatient medical center, an emergency shelter during winter season (November to March), a clothing store, a counseling service for people with unclear health insurance coverage (Clearingstelle), and a temporarily established 24/7 shelter.

Funded by the Berlin-Mitte district, the Senate Department for Integration, Labor and Social Affairs, and the Senate Department for Finance, the facility provided the only opportunity for isolation and quarantine for PEH in Berlin during the period of time described.

Patients were admitted from the entire city area under the coordination of the local health authority. Bed capacity of the facility fluctuated over time due to room arrangements and capacity expansion. From December 2020 to June 2021, the bed capacity ranged from 16 to 70 beds for confirmed SARS-CoV-2 infected PEH and between 14 and 30 beds for suspected cases or close contacts. Due to limited human resources, it was not always possible to fully utilize the bed capacity. Between 10 and 32 staff members worked at the facility, building a multilingual (German, Polish, Russian, Spanish, English, Romanian, Arabic, and Indonesian), interdisciplinary team consisting of medical professionals (physicians, nurses, medical students), social workers, and volunteers. The staff was available 24/7 for the patients providing daily meals and snacks, hygiene kits, help with the maintenance of the rooms, and other services. Medical care was provided by the medical professionals including daily monitoring of vital parameters and treatment of COVID-19 symptoms and other underlying health conditions. Admission of drug users was enabled through close cooperation with physicians specialized in opioid substitution therapy (OST). In case of reported substance use, a consultation with these physicians took place with regular evaluation and adjustment of OST if necessary. For patients already in OST, substitution was continued according to the treating physician’s recommendations. For alcohol dependent patients, individualized rations of alcohol (beer, vodka, wine) were provided and adjusted daily if needed based on previous daily consumption, alcohol breath testing and withdrawal symptoms. Tobacco was provided on demand and patients were allowed to consume tobacco in designated areas.

Patients were supported in social and bureaucratic issues where needed, e.g., clarification of financial support from the state, housing after the isolation/quarantine period, obtaining identity documents, or access to state subsidized health insurance. Pastoral care or psychological counseling was provided on request. Additional offers included storage of the patients’ belongings, TV, free Wi-Fi, table games, books, newspapers, and others.

Patients were housed in shared bedrooms. Within the isolation unit, patients could move freely and shared the common area, the kitchen, the smoking area, and the balconies.

### 2.2. Data description and sources

The data was not collected for study purposes, but as part of the daily routine of the isolation facility. Subsequently (retrospectively), the data was extracted for the purpose of this study.

Upon arrival, name, date of birth, spoken languages, pre-existing health conditions, medication, SARS-CoV-2 symptoms, substance use, and dietary needs were registered in the patient’s records. SARS-CoV-2 test results, prescribed duration of the isolation period by the local health authority and reasons for premature discontinuation or longer stay were documented.

Medical staff recorded symptoms, body temperature (with an infrared forehead thermometer), heart rate, oxygen saturation (SpO2) and blood pressure on a daily basis, as well as medication needs. Staff-observed psychological distress, required psychological or social counseling or other special needs were recorded in a non-standardized way.

### 2.3. Measures

We analyze socio-demographic patient characteristics including age, sex, spoken languages, existing health conditions and substance use.

Probable COVID-19 related reasons for hospitalization (low oxygen saturation, dyspnea) were differentiated from non-COVID-19 related reasons. The cases hospitalized due to COVID-19 progression are described in more detail.

The primary outcome of this study was completion of the isolation period. The secondary outcome was associated factors with completion or non-completion of isolation period. The isolation period was considered not completed, if: (i) the period was terminated against medical advice; (ii) the patient was discharged from the facility for behavioral reasons; and (iii) the period was temporarily interrupted against medical advice. Patients who were referred to a hospital were not included in the definition of not completing the isolation period.

COVID-19 related symptoms were defined according to the RKI (29), and non-COVID-19 related medical needs were grouped into nine categories (hypertension, withdrawal symptoms, musculosceletal conditions, urogenital diseases, sleeping disorders, dermatological conditions, staff-observed psycological distress, wound management, others). High blood pressure was defined according to the 2018 ESC/ESH guidelines (30).

### 2.4. Data analysis

Data were retrospectively extracted by a study physician from the paper-based and additional electronic records. We used descriptive statistics, including counts, proportions, means, and standard deviations, to summarize characteristics accordingly. Potential association of completing isolation with age, sex, smoking status, consumption of alcohol and opioids, and language barrier were assessed by Student’s *t*-test (age) or Fisher’s exact test. A value of *p* below 0.05 was considered statistically significant. We used R version 4.1.2 for all analyses.

### 2.5. Ethics statement

This study was approved by the ethics committee of the Charité – Universitätsmedizin Berlin (No.: EA2/162/21).

## 3. Results

### 3.1. Demographic characteristics

We included 139 PEH with PT-PCR confirmed SARS-CoV-2 infection in the study. Two individuals were admitted twice with a confirmed reinfection, resulting in a total of 141 admissions. The sociodemographic and health characteristics are shown in Table 1. Mean age was 45.4 years (standard deviation [SD], 13.1); the majority (88.5%) were male. Among pre-existing health conditions, dermatological conditions (12.9%), cardiovascular disease (8.6%), musculoskeletal and psychiatric conditions (each 7.2%) were stated most frequently (Table 1). The majority (76.5%) of the patients were current smokers, nearly half (46.3%) reported regular alcohol consumption, and 16.5% used opioids, of which half were already in OST prior to admission while the other half initiated OST at the isolation facility.

**Table 1:** Characteristics of 139 people experiencing homelessness with RT-PCR confirmed SARS-CoV-2 infection admitted to the isolation facility.

Characteristics	% (n/N)
Age	Mean (SD) 45.4 (13.1)
Sex	
Male	88.5% (123/139)
Female	11.5% (16/139)
Comorbidities according to medical history	41% (57/139)
Malignancy	0.7% (1/139)
Cardiovascular disease	8.6% (12/139)
Respiratory system disease	5.8% (8/139)
Chronic liver disease	2.2% (3/139)
Infectious disease	5% (7/139)
Metabolic disease	4.3% (6/139)
Neurological disease	5% (7/139)
Musculoskeletal condition	7.2% (10/139)
Psychiatric condition	7.2% (10/139)
Dermatological condition	12.9% (18/139)
Others	6.5% (9/139)
Tobacco consumption^1^	76.5% (101/132)
Alcohol consumption^1^	46.3% (63/136)
Opioid use	16.5% (23/139)
Pre-existing opioid substitution therapy	7.9% (11/139)
Opioid substitution therapy initiated	8.6% (12/139)
Spoken languages	
German	59% (82/139)
English	8.6% (12/139)
Russian	17.3% (24/139)
Polish	24.5% (34/139)
Romanian	4.3% (6/139)
Bulgarian	5.8% (8/139)
Others	20.9% (29/139)
Language barrier documented	18% (25/139)

1 Missing data *n* = 7 and *n* = 2, respectively.

The most common spoken languages were German (59%), Polish (24.5%), Russian (17.3%), English (8.6%), Bulgarian (5.8%), and Romanian (4.3%), whereby some patients reported being multilingual. In 18% of the cases, the team documented a language barrier meaning that communication between staff and patient was complicated.

### 3.2. Clinical characteristics

In 81% of the admissions, patients were symptomatic, with a median duration of symptoms [range] of 5 days [1–26]. The most commonly reported symptoms (Table 2) affecting approximately half of the patients were cough, rhinorrhea, and headache. Ten to 20% of patients complained about sore throat, gastrointestinal symptoms, myalgia, shortness of breath, and/or loss of smell and/or taste (Table 2). Fever (>38°C) was observed in four patients. By daily screening of SpO2, one in three patients (32.6%) at least once during their stay showed an oxygen saturation below 95%; seven patients (5%) dropped below 90%.

**Table 2:** Clinical characteristics of 139 people experiencing homelessness with RT-PCR confirmed SARS-CoV-2 infection admitted to the isolation facility.

Clinical characteristics	% (n/N)^1^
Symptomatic	80.9% (114/141)
Asymptomatic	19.1% (27/141)
Duration of symptoms	Median [Range] 5.0 [1.0–26.0]
Reported symptoms	
Cough	58.9% (83/141)
Rhinorrhea	54.6% (77/141)
Myalgia	12.8% (18/141)
Headache	45.4% (64/141)
Gastrointestinal	14.9% (21/141)
Sore throat	16.3% (23/141)
Shortness of breath	11.3% (16/141)
Loss of smell and/or taste	9.9% (14/141)
Others	19.1% (27/141)
Fever (>38°C)	2.8% (4/141)
SpO2 < 95%	32.6% (46/141)
SpO2 < 90%	5% (7/141)
Other medical conditions during stay	80% (113/141)
Hypertension Grade 1	29.1% (41/141)
Hypertension Grade 2	19.9% (28/141)
Hypertension Grade 3	10.6% (15/141)
Withdrawal symptoms	15.6% (22/141)
Musculosceletal conditions	9.9% (14/141)
Urogenital diseases	4.3% (6/141)
Sleeping disorders	8.5% (12/141)
Dermatological conditions	14.2% (20/141)
Staff-observed psychological distress	19.9% (28/141)
Wound management	12.8% (18/141)
Others	16.3% (23/141)
Hospitalization	8.5% (12/141)
Hospitalization due to COVID-19 progression	2.1% (3/141)
Hospitalization due to COVID-19 progression	6.4% (9/141)
Specialized outpatient medical care	2.8% (4/141)

1 Clinical characteristics of 139 individual patients. Two individuals were admitted twice with an assumed reinfection, resulting in a number of *N* = 141 clinical observations.

Arterial hypertension was the most common (59.6%) non-COVID-19 related condition observed. As only 12 patients reported a cardiovascular disease as a pre-existing health condition, in most cases hypertension was unknown prior to isolation. Withdrawal symptoms were seen in 16% of patients and 20% were affected by staff-observed psychological distress symptoms (e.g., depression, delusion, auditory hallucinations, symptoms of post-traumatic stress disorder or schizophrenia, anxiety and panic attacks, disorientation and confusion, aggression). Dermatological conditions, e.g., rash, itching, ulcers, psoriasis, abscesses or wounds were seen in 14% (Table 2).

During the study period, three patients were hospitalized due to COVID-19 progression and SpO2 < 90%. These were two women and one man aged 58, 59, and 77 years, respectively. All had a hypertensive crisis with systolic blood pressure of >200 mmHg at some point during their stay at the isolation facility. Additionally, one patient had type 2 diabetes mellitus, and another had a newly diagnosed mamma carcinoma. All were transferred back to the isolation facility after their hospital stay. No deaths were reported. Details of treatment during hospitalization were not available for analysis in this study.

Nine other patients were hospitalized for non-COVID-19 related medical health conditions: fractures, uncontrolled hyperglycemia, ophthalmological emergency, hypertensive crisis, suspected case of neurosyphilis, pyelonephritis, opioid overdose (due to simultaneous consumption of heroin besides the OST) and an untreated HIV-infection with a suspected opportunistic infection.

Specialized outpatient care was needed for four patients due to lower abdominal pain, thoracic pain, alcohol withdrawal, and agitation. All returned to the isolation facility after treatment.

### 3.3. isolation related aspects

The majority of patients (80.7%) were referred from a PEH service provider to the isolation facility (Table 3). Reasons for referral were a positive antigen or RT-PCT test result. The median length of stay at the isolation facility was 2 weeks. Extended isolation periods were due to persistence of symptoms or due to continued positive antigen test results.

**Table 3:** Isolation related aspects of 139 people experiencing homelessness with RT-PCR confirmed SARS-CoV-2 infection admitted to the isolation facility.

Characteristics	% (n/N)^1^
Length of stay	Median [Range] 14.0 [2.0, 41.0]
Prolongation of isolation	16.3% (23/141)
Source of referral^2^	
Homeless service provider or shelter	80.7% (96/119)
Hospital	8.4% (10/119)
Outpatient	2.5% (3/119)
Others	8.4% (10/119)
Reason for referral^2^	
Positive rapid antigen test	84.6% (110/130)
Positive RT-PCR test	15.4% (20/130)
Isolation completed	81.6% (115/141)
Isolation not completed	18.4% (26/141)
Interruption against medical advice	8.5% (12/141)
Termination against medical advice	7.8% (11/141)
Discharge for behavioral reasons	2.1% (3/141)
Use of social assistance	65.2% (92/141)

1 Clinical characteristics of 139 individual patients. Two individuals were admitted twice with an assumed reinfection, resulting in a number of *N* = 141 clinical observations.

2 Missing data *n* = 22 and *n* = 11, respectively.

In total, 115 patients completed their required isolation period. Of the 26 patients not completing isolation, 11 terminated their stay themselves, three were discharged for behavioral or safety reasons, and 12 interrupted their stay but returned.

During the isolation period, almost half of the patients required managed alcohol substitution and one in six OST. Two thirds of the patients used the social counseling service.

Participants who did not complete their isolation period tended to be younger than those who did (mean age [SD], 42.6 [12.8] vs. 46.0 [13.1] years, *p* = 0.2). The percentage of female patients who did not complete their isolation period was with 7.7% (2/26) slightly less compared to 12.4% (14/113, *p* = 0.7) who completed it. Regular consumption of alcohol was reported by 52.0% (13/25) in the non-compliant group, versus 45.0% (50/111, *p* = 0.5) in the compliant group, whereas for opioid use these numbers were 15.4% (4/26) versus 16.8% (19/113, *p* = 0.9). A language barrier among patients who did not complete their isolation was documented for 7.7% (2/26) compared to 20.4% (23/113, *p* = 0.1) among those who completed their isolation period.

## 4. Discussion

This study aimed to gain insight into a COVID-19 isolation facility for PEH in Berlin between December 2020 and June 2021. A high completion rate of the isolation period has been achieved. Based on the services used and the patient’s health condition we draw conclusions on special needs of the patients and requirements for future facilities.

### 4.1. Demographic and clinical characteristics

The mainly male and middle-aged study population was characterized by a high proportion of comorbidities. In contrast to other studies on PEH, we observed considerably less cardiovascular diseases (8.6%) compared to California/USA (61.5%) and Berlin/Germany (17–37.2%), as well as less respiratory diseases (5.8%) than in Ontario/Canada (24.9% for asthma and COPD) (10, 16, 31). Differences may be explained by a possible underestimation of comorbidities in our study as they were self-reported and documented based on the medical history. The proportion of tobacco smokers (77%) in our study population greatly exceeds the proportion in the general population in Germany (30%) (32), but resembles observations in other international studies on PEH (14, 31, 33). With 46%, the share of PEH regularly consuming alcohol is higher than in other studies (37%) (18) but consistent with a systematic review that found higher prevalence rates of alcohol use disorders in German PEH (17, 18). In our study, 16.5% of the patients received OST which is comparable to a similar program at a facility in Canada (22%) (33). However, the rate of drug use are difficult to compare because they vary in heterogeneous regions and milieus (18). A systematic review focusing on PEH in four German cities found a prevalence of drug use among PEH of 14% (17).

In 81% of the 141 admissions, COVID-19 related symptoms were reported. Three patients were hospitalized due to COVID-19 progression, resembling a similar hospitalization rate in an isolation and quarantine facility for PEH in San Francisco (34). We did not assess associations of patients´ characteristics with hospitalization due to the low number of hospitalized PEH. Even though the proportion of asymptomatic infections in our study (19%) is similar to other data among PEH in Atlanta, Georgia in 2020 (35), these figures must be interpreted cautiously. Withdrawal symptoms might overlap with COVID-19 symptoms which makes differentiation difficult (1), and we cannot exclude that positive but asymptomatic PEH decided not to isolate themselves.

Details on non-COVID-19 related medical conditions and hospitalizations provide important information on the general health status of PEH and their medical needs. General medical conditions were common in the present study including more than half of the patients having arterial hypertension. The high proportion of uncontrolled hypertension accords with other findings among PEH (14) and is considerably increased in PEH compared to the general population. Also, the high number of non-COVID-19 related hospitalizations reflects a poor health status of PEH included in this study.

### 4.2. Isolation related aspects

Compliance with completing the isolation period was high (81.6%) with a median duration of the isolation period of 2 weeks. The variability of stay duration in the facility (2–41 days) reflects premature termination of isolation and persistence of SARS-CoV-2 positivity. Twelve patients interrupted their required isolation period but returned to the facility to continue isolation. They were categorized as non-completers which explains their higher proportion (18.4%) compared to other studies describing similar programs, that did not include short-time interruption of isolation period to their assessment (33, 34). Reasons for discontinuation of isolation are multifactorial and may depend on program design, staff-patient relations, availability of alcohol, nicotine, and opioid replacement program as well as on individual experiences with the health system that has led to trust or distrust. In this context, Fuchs et al. emphasize the importance of maintaining good communication with patients explaining the reasons and importance of quarantine or isolation. Further qualitative investigations in this context should be encouraged (34).

We did not observe a statistically significant association between completion of the isolation period and assessed characteristics. Elsewhere premature discontinuation has been attributed to female gender and young age (34). Unsheltered PEH and those who went to quarantine because of a close contact to a positive case had greater odds of premature discontinuation (34); as these variables were not under investigation in our study, we cannot compare them. Both, this, and our study, did not find different alcohol and opioid use between the groups, indicating that PEH with substance use can successfully finish an isolation period with an appropriate program for OST or controlled alcohol distribution in place.

### 4.3. Limitations

The study is limited by its retrospective nature. Due to temporary shortage of staff and a high workload in the daily routine, the documentation may be incomplete in some cases. Certain parameters, like clinical characteristics, documented language barriers or use of social support, may be underreported.

Our study population is unlikely to represent the total population of roofless PEH in Berlin. As most patients were referred from another homeless service provider, the study population mainly includes PEH with access to these services and who agreed to be referred to the isolation facility. People living in hidden homelessness or those with no access to the support systems were not reached. Overall figures for Berlin regarding PEH tested for or infected with COVID-19 are not existing. Thus, the results of the study cannot be put into relation.

Due to missing information during the hospitalization period, the severity of the COVID-19 infection of the hospitalized patients could not be analyzed more precisely, which allows only a limited statement about the course of the disease.

Our descriptive study was not designed to assess differences between those who completed isolation and those who did not. Due to the low patient numbers these results have to be interpreted cautiously.

Large heterogeneity exists in PEH. Homelessness is a complex interplay between individual, interpersonal, and socioeconomic factors (36). To better reflect this heterogeneity, specific determinants would have to be assessed, e.g., detailed living and working situation, residential status, insurance status, as well as experienced discrimination (direct, institutional, and structural).

### 4.4. Needs and requirements for an isolation facility for PEH

Internationally, there is only a small number of studies on the specific needs and requirements of an isolation facility for PEH. This study is the first to analyze such a facility in Germany. Our findings indicate a high demand for a managed alcohol program and OST in a COVID-19 isolation facility. Studies on programs without OST reported challenges with withdrawal symptoms and consequently with the adherence to the quarantine or isolation period (37, 38). An emergency safe supply drugs and managed alcohol program in a COVID-19 isolation hotel for PEH in Canada achieved high rates of successful completion of isolation and low rates of adverse events (33).

Professional psychological care onsite appears important, as referral for mental health care with concomitant COVID-19 infection can be difficult to realize. In our study population, psychological distress was observed by the staff in almost 20% of the patients, demonstrating a substantial need for psychological care. However, there is an unmet need for adequate mental health care of PEH that can be particular challenging during isolation (39). Mental disorders have been perceived a major challenge in a similar study (34).

A high number of patients showed non-COVID-19 related medical conditions that required care during their stay in the isolation facility. Due to various system related and individual obstacles, it was often difficult to ensure further treatment of medical conditions after the isolation period (informal communication with staff). This may indicate the presence of multiple structural barriers of PEH in Germany to the regular health care system and emphasizes the need for better access.

A crucial function of the isolation facility was providing safe shelter for multiple days and nights. Safe shelter is known not only to reduce the rate of COVID-19 infections but also to improve the general health status and to allow self-determination (40, 41). In our study, 65% of patients used the opportunity to consult support services for social and bureaucratic issues. Although not documented in a standardized way, referral to 24/7 residential home was arranged for some cases. This illustrates the important additional benefit of integrated social services into isolation facilities.

Even though several languages were covered by the multilingual team in the isolation facility, language barriers were documented for 18% of the patients. To address this, language mediation over a telephone was used for the languages that could not be provided. Communication with appropriate language mediation is important to explain the process and meaning of the isolation and to gain trust and compliance with the measures.

After the end of the study in June 2021, the isolation facility enabled the admission of further 285 PEH (personal communication). The project duration of the central isolation facility described in this study was extended several times at very short notice, until it was closed at the end of the winter season in April 2022. Even though alternative offers were provided in decentralized settings, lack of isolation capacity and difficulties in the provision of shelter for SARS-CoV-2 infected PEH were subsequently reported (42, 43). The provision of adequate professional medical expertise during isolation, including OST, can be difficult in decentralized settings.

### 4.5. Recommendations for future research

There is little consistent data on PEH in Germany. In particular, health-related data on PEH are scarce and, as in this case, are often based on routine data collected in low-threshold facilities. Existing data are usually not standardized and cannot be compared with each other. A survey with standardized, uniform data sets would be desirable and could provide a more holistic insight into the health situation and needs of PEH in Germany. In addition, close cooperation between practice and research is essential to advance research on homelessness and health.

Qualitative interviews to assess the living conditions of PEH and their needs as well as their motivations for or against accepting offers of medical services would be a relevant addition to the data presented and could provide a more holistic picture.

## 5. Conclusion

The isolation facility for PEH was an essential component of COVID-19 prevention and control at the time of the study. It enabled completion of isolation and provided access to adequate medical care. Despite a high rate of comorbidities, hospitalization rate due to COVID-19 was low. PEH with substance or alcohol use could successfully finish isolation with an appropriate program for substitution in place. Based on the utilization of services, we conclude that language mediation, psychological care and support from social workers are important components of such a facility. Moreover, the isolation facility provided safe shelter for a certain period of time and an opportunity to address non-COVID-19 related medical and social conditions. The positive effects of the provision of safe shelter for PEH combined with social and medical services advocate for a similar approach for other health conditions.

## Data availability statement

The datasets generated and analyzed during the current study are not publicly available but are available from the corresponding author on reasonable request.

## Ethics statement

The studies involving human participants were reviewed and approved by the Ethikkommission der Charité – Universitätsmedizin Berlin, Charité – Universitätsmedizin Berlin, corporate member of Freie Universität Berlin and Humboldt Universität zu Berlin. Written informed consent for participation was not required for this study in accordance with the national legislation and the institutional requirements.

## Author contributions

MH: conceptualization, methodology, investigation, formal analysis, and writing original draft. SK and SK-N: investigation and conceptualization. WL: formal analysis and scientific advice. LM, RR, and CS: conceptualization and supervision. AS: conceptualization and scientific advice. GE, RZ, FM, and JS: scientific advice and supervision. AL and NS: conceptualization, methodology, formal analysis, and scientific advice. MH, SK, SK-N, WL, LM, RR, CS, AS, GE, RZ, FM, JS, AL, and NS: writing—review and editing. All authors contributed to the article and approved the submitted version.

## Conflict of interest

The authors declare that the research was conducted in the absence of any commercial or financial relationships that could be construed as a potential conflict of interest.

## Publisher’s note

All claims expressed in this article are solely those of the authors and do not necessarily represent those of their affiliated organizations, or those of the publisher, the editors and the reviewers. Any product that may be evaluated in this article, or claim that may be made by its manufacturer, is not guaranteed or endorsed by the publisher.
